# A nutrient mediates intraspecific competition between rodent malaria parasites *in vivo*

**DOI:** 10.1098/rspb.2017.1067

**Published:** 2017-07-26

**Authors:** Nina Wale, Derek G. Sim, Andrew F. Read

**Affiliations:** 1 Center for Infectious Disease Dynamics and Department of Biology, The Pennsylvania State University, University Park, PA 16802, USA; 2 Department of Entomology, The Pennsylvania State University, University Park, PA 16802, USA

**Keywords:** resource competition, within-host dynamics, co-infection, malaria, *Plasmodium chabaudi*, disease

## Abstract

Hosts are often infected with multiple strains of a single parasite species. Within-host competition between parasite strains can be intense and has implications for the evolution of traits that impact patient health, such as drug resistance and virulence. Yet the mechanistic basis of within-host competition is poorly understood. Here, we demonstrate that a parasite nutrient, para-aminobenzoic acid (pABA), mediates competition between a drug resistant and drug susceptible strain of the malaria parasite, *Plasmodium chabaudi*. We further show that increasing pABA supply to hosts infected with the resistant strain worsens disease and changes the relationship between parasite burden and pathology. Our experiments demonstrate that, even when there is profound top-down regulation (immunity), bottom-up regulation of pathogen populations can occur and that its importance may vary during an infection. The identification of resources that can be experimentally controlled opens up the opportunity to manipulate competitive interactions between parasites and hence their evolution.

## 1. Introduction

Hosts are often infected by multiple parasite ‘strains’—parasites of the same species that have a different genotype and, often, phenotype [1,2]. In the last decades, evidence for within-host competition between parasite strains, including those of the causative agents of malaria and sleeping sickness, has accumulated (e.g. [3–9]) and interstrain competition has been implicated as a driver of the evolution of virulence [10,11], antigenic diversity [12,13] and drug resistance [14,15]. As recognition of the role that within-host competition plays in the dynamics and evolution of parasite populations has increased, so too has interest in harnessing it for the control of parasite populations [16,17]. Yet which aspects of the within-host environment mediate competition between parasite strains is still poorly understood, limiting our ability to study the role that competitive interactions play in parasite ecology and evolution and to manipulate these interactions to our advantage.

Parasites can compete through direct, aggressive interactions or indirectly through common enemies, such as immune cells, or via the consumption of shared resources, such as space and nutrients. What mediates intraspecific competition has consequences for disease at both the level of the individual host [11] and at the population level, since the mechanism of competition may impact the evolution of virulence [18]. While theoretical studies posit a role for all three mechanisms of interstrain competition [19–21], only the role of immune-mediated apparent competition (e.g. [9,22–25]) and interference competition (e.g. [26]) have, so far as we are aware, been investigated empirically *in vivo*.

Here, we demonstrate that a nutrient mediates competition between strains of the rodent malaria parasite *Plasmodium chabaudi* in mice with a fully intact immune system and investigate the consequences of resource abundance and the intensity of within-host competition for host health. *Plasmodium* parasites require folate for pyrimidine synthesis and methionine metabolism [27] and, unlike their mammalian hosts who acquire folate from their diet, are able to synthesize it from 6-hydroxymethyl-7,8-dihydropterin pyrophosphate and para-aminobenzoic acid (pABA) [28]. *In vivo*, pABA limits the growth of several species of malaria parasites [29–33], including the rodent malaria parasite *P. chabaudi*, which is used as a model of human malaria infection. As a result, experimental animals in studies of *P. chabaudi* are routinely supplemented with pABA [34,35]. Here, we investigate the impact of pABA concentration on the intensity of competition between two genetically distinct strains of *P. chabaudi*, AJ and AS_pyr_. Competition between these strains is well characterized and asymmetrical: in mice inoculated with the two strains at the same time, AJ strongly suppresses AS_pyr_; AS_pyr_ has little to no effect on AJ [4,5,36,37]. The pABA requirements of AJ and AS_pyr_, by contrast, have not been studied. We show that the intensity of competition between these strains of *P. chabaudi* in the period before they are cleared by the immune system varies over a gradient of pABA supply and that resource availability changes the relationship between parasite burden and pathology.

## 2. Material and methods

Hosts were female six- to eight-week-old C57BL/6 J mice, maintained on 5001 Laboratory Rodent Diet (LabDiet, USA). pABA was administered to mice via drinking water at a concentration of 0.05% (high treatment), as is standard in experiments involving rodent malaria [34,35], 0.01% (medium treatment), 0.005% (low treatment) or 0% (unsupplemented treatment). Ten mice were assigned to each pABA treatment: five were infected by intraperitoneal injection with 10^6^ parasites of the pyrimethamine resistant AS44p strain (hereafter, AS_pyr_), five with 10^6^ AS_pyr_ and 10^6^ parasites of the pyrimethamine susceptible AJ strain for a total of eight treatments, each containing five mice as replicates (table 1). We used a higher total density of parasites in the competition treatment (i.e. an additive experimental design) because we wanted to determine the change in performance of a focal strain (AS_pyr_) when a competitor (AJ) is present [38].

**Table 1.** Number of mice in each experimental treatment.

	pABA treatment			
	high	medium	low	unsupplemented
single infections (AS_pyr_ alone)	5 (1^a^)	5	5 (1^a^)	5
mixed infections (AJ + AS_pyr_)	5 (5^b^)	5	5 (1^a^)	5

^a^Mice removed from all analyses as they were inoculated with fewer parasites than intended.

^b^Mice died. The dynamics of infections of each individual mouse can be found in the electronic supplementary material, figures S1 and S2.

Infections were monitored daily from days 3 to 21 post-inoculation (PI), the period during which parasites are consistently detectable by quantitative PCR (qPCR) [36]. Each day, 7 µl of blood was taken from the tail: 2 µl for the immediate quantification of red blood cell (RBC) density via flow cytometry (Beckman Coulter) and 5 µl for the quantification of parasite density by qPCR, using methods previously described [39,40]. The 5 µl blood sample was centrifuged at 13 000*g* for 1 min, the supernatant removed, and the remaining blood pellet stored in citrate saline at −80°C, prior to analysis. As an additional measure of morbidity, mouse weight was measured. Experiments were conducted in accordance with the protocol approved by the Institutional Animal Care and Use Committee of the Pennsylvania State University (permit number 44512).

Statistical analysis was performed using R [41]. For each mouse, we calculated total parasite density, total RBC density and total weight, the cumulative sum of these measures over time. *Plasmodium chabaudi* parasites reproduce synchronously every 24 h, so that integrating across time gives the total number of parasites produced during that time period. All measurements of parasite density were log_10_ transformed prior to analysis. Since the variance in total parasite density changed systematically with pABA treatment, we analysed total parasite density using generalized least-squares (GLS) models with pABA treatment specified as a variance covariate, following [42]. The temporal dynamics of infections were analysed using linear mixed effects (LME) models following [42–44], with day fitted as a factor to allow for nonlinearity in infection dynamics, individual mouse fitted as a random effect, a corAR1 autocorrelation structure fitted to correct for temporal autocorrelation and a variance structure that accounted for changes in residual variance in parasite density between days. To tease apart the effect of pABA on the dynamics of competition, post hoc analysis was performed using the *lsmeans* package [45]. The impact of pABA on the growth rate of the parasite population was similarly analysed. Since the growth rate of parasite populations in some mice had slowed by the fifth day, only data from days 3 and 4 were used for the analysis of initial replication rate. Both GLS and LME models were fitted using the *nlme* package [46]. Model simplification was performed by sequentially dropping the least significant term, until all terms were significant. Least significant terms were identified using likelihood ratio tests, for GLS and LME models, and *F*-tests for standard linear regression models.

Three mice received a smaller number of parasites than was intended and were removed from all analyses (table 1; electronic supplementary material, figures S1 and S2). All mice in the high pABA mixed infection treatment eventually succumbed to infection and day 8 was the last day on which all mice were alive. The effect of pABA concentration on the magnitude of competitive suppression was therefore assessed during the period between days 3 and 8 PI, using data from all treatment groups, and then again during the period from days 3 to 21 PI, with the high pABA treatment excluded.

## 3. Results

### (a) Parasite dynamics in single infections

**Figure 1.**
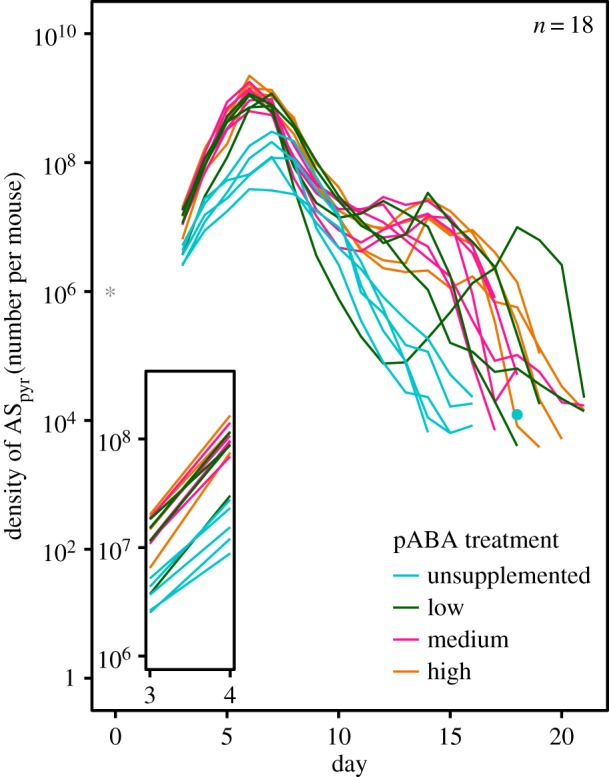
pABA is a limiting resource for AS_pyr_. Infection dynamics of single infections of AS_pyr_ in unsupplemented (blue), low (green), medium (pink) and high (orange) pABA treatments. Each line represents the dynamics of infection in an individual mouse. *n* specifies the number of mice plotted and included in the analysis. The star represents the number of parasites that were inoculated and the time at which they were administered; the dot the density of parasites detected in an instance when parasites were not detected the day before or after. Inset shows the infection kinetics between days 3 and 4.

In mice infected with AS_pyr_ alone_,_ pABA supplementation increased both the growth rate and total size of AS_pyr_ infections (figure 1; electronic supplementary material, figure S3 solid circles, *total density* pABA 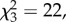 *p* < 0.001, *growth rate* 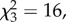 *p* = 0.001). AS_pyr_ grew almost twice as fast in the high pABA treatment as in the unsupplemented treatment. The kinetics of infections were also altered by pABA treatment (figure 1, *parasite density* day × pABA 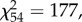 *p* < 0.001). After their peak, parasite densities declined continuously in the unsupplemented treatment; by contrast, in the pABA supplemented treatments the density of AS_pyr_ increased or plateaued during the post-peak phase, causing a hump in the infection dynamics.

### (b) Parasite dynamics in mixed infections

**Figure 2.**
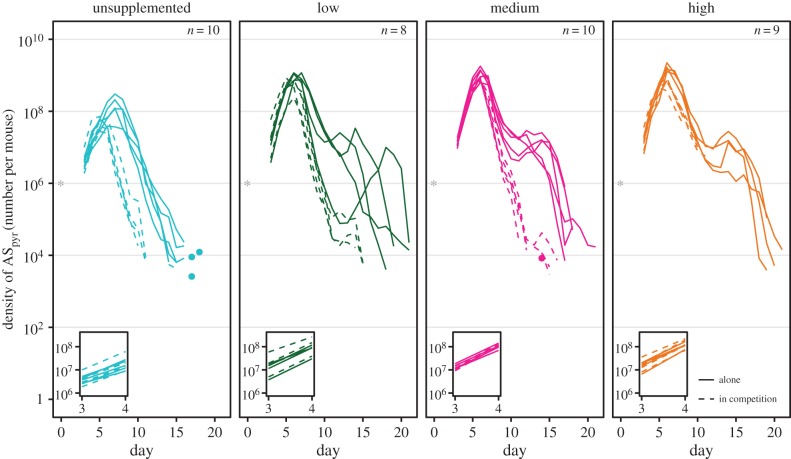
pABA mediates competition between AS_pyr_ and AJ. Dynamics of AS_pyr_ alone (solid lines) and in competition with AJ (dashed lines) in unsupplemented (blue), low (green), medium (pink) and high (orange) pABA treatments. Each line represents the dynamics of infection in a single mouse. The dynamics of AS_pyr_ in competition in the high pABA treatment (dashed, orange lines) are attenuated because the mice died. Note that, in mixed infections, AJ was detectable until the end of the time series in the majority of mice (see the electronic supplementary material, figures S2 and S4). *n* specifies the number of mice plotted and included in the analysis of parasite dynamics. Stars represent the number of parasites inoculated and the time at which they were administered. Dots indicate the density of parasites detected on a particular day in instances where parasites were not detected the day before or after. Insets show the infection kinetics between days 3 and 4. (Online version in colour.)

In mice with mixed infections, AS_pyr_ was competitively suppressed by AJ, irrespective of pABA treatment (figure 2; electronic supplementary material, figure S3). Both the dynamics and intensity of competition varied with pABA treatment in the period when all mice were alive (figure 2; *parasite density* day × pABA × competition 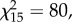 *p* < 0.001), though the significance of pABA's impact on the intensity of competition, as measured by the change in total infection size in mixed versus single infections, was sensitive to the inclusion of a particular mouse (electronic supplementary material, figure S3 days 3–8, *total density* pABA × competition excluding ‘outlier’ 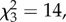 *p* < 0.01; including ‘outlier’ 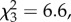 *p* = 0.09). This mouse apparently received fewer parasites than its treatment-mates (electronic supplementary material, figure S2). AJ did not impact the initial growth rate of AS_pyr_ (figure 2, inset *growth rate* competition 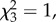 *p* = 0.8) but shortened the time it took for AS_pyr_ to reach its peak density and reduced the peak's magnitude, most markedly in the unsupplemented treatment (figure 2). AS_pyr_ experienced significant competitive suppression in the unsupplemented and high pABA treatments sooner than in the low or medium treatments (day 6 versus day 7, figure 2). In this initial phase of the infection, competitive suppression was most intense in the high pABA treatment (electronic supplementary material, figure S3).

The dynamics of competition continued to be impacted by pABA supplementation in the mice that survived (figure 2; days 3–21 *parasite density*, day × pABA × competition 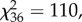 *p* < 0.001; *total density* pABA × competition 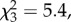 *p* = 0.07). Between days 7 and 9, when the density of AS_pyr_ was declining in all treatments, competitive suppression was strongest in the unsupplemented treatment. AS_pyr_ was competitively excluded by AJ sooner in the unsupplemented treatment than in the supplemented treatments (figure 2). In the supplemented treatments, the presence of AJ resulted in the disappearance of the post-peak hump that was observed in single infections.

The size of AJ infections in mice with mixed infections was unaffected by pABA supplementation (electronic supplementary material, figure S4, *total density days 3–8* pABA *F*_3,15_ = 1.7, *p* = 0.2, *total density days 3–21 F*_2,11_ = 1, *p* = 0.4).

### (c) Virulence of infections

**Figure 3.**
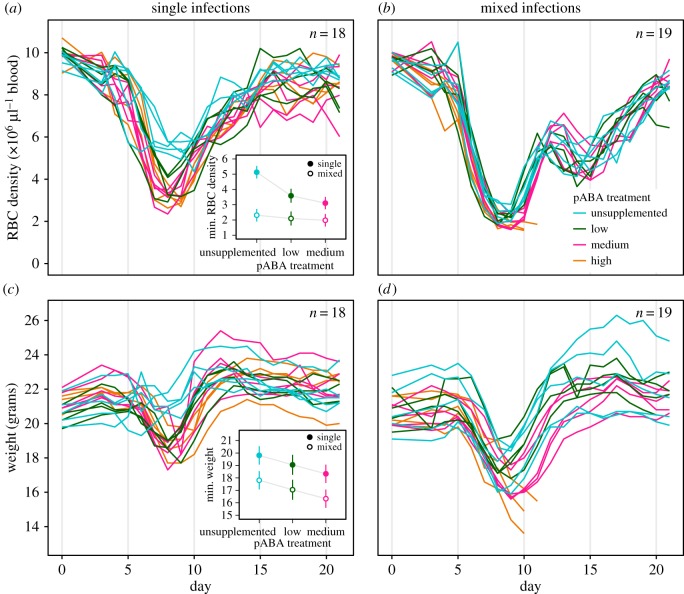
pABA supplementation increases the severity of disease. Dynamics of red blood cells (RBCs) (*a*,*b*) and weight (*c*,*d*) in individual mice infected with AS_pyr_ alone (*a*,*c*) or with both AS_pyr_ and AJ (*b*,*d*) and given unsupplemented (blue) water or water supplemented with a low (green), medium (pink), or high (orange) concentration of pABA. Insets show the best-fit model estimates of the mean minimum RBC density (*a*, inset) and minimum weight (*c*, inset) of mice in the single infection (filled circles) and mixed infection (open circles) treatments. Error bars show the 95% confidence intervals around these estimates. *n* specifies the number of mice plotted and included in the analysis.

Disease severity was worsened by pABA supplementation, particularly in singly-infected mice (figure 3). Disease was more severe in mice with mixed infections of AS_pyr_ and AJ (figure 3) and especially so in the high pABA treatment, where all of the mice died (table 1). In the remaining treatments, pABA supplementation worsened anaemia in singly-infected mice but not mice co-infected with AS_pyr_ and AJ (figure 3*a*,*b*, *total RBC density* pABA × competition *F*_2,22_ = 6.4, *p* < 0.01; *minimum RBC density*; *F*_2,22_ = 10, *p* < 0.001). Mice supplemented with pABA experienced more acute weight loss, irrespective of whether they were co-infected (figure 3*c*,*d minimum weight* pABA × competition *F*_2,22_ = 2.7, *p* = 0.09, *minimum weight* pABA *F*_2,24_ = 6, *p* < 0.01), but they did not lose more weight overall (*total weight* pABA *F*_2,24_ = 0.8, *p* = 0.5).

**Figure 4.**
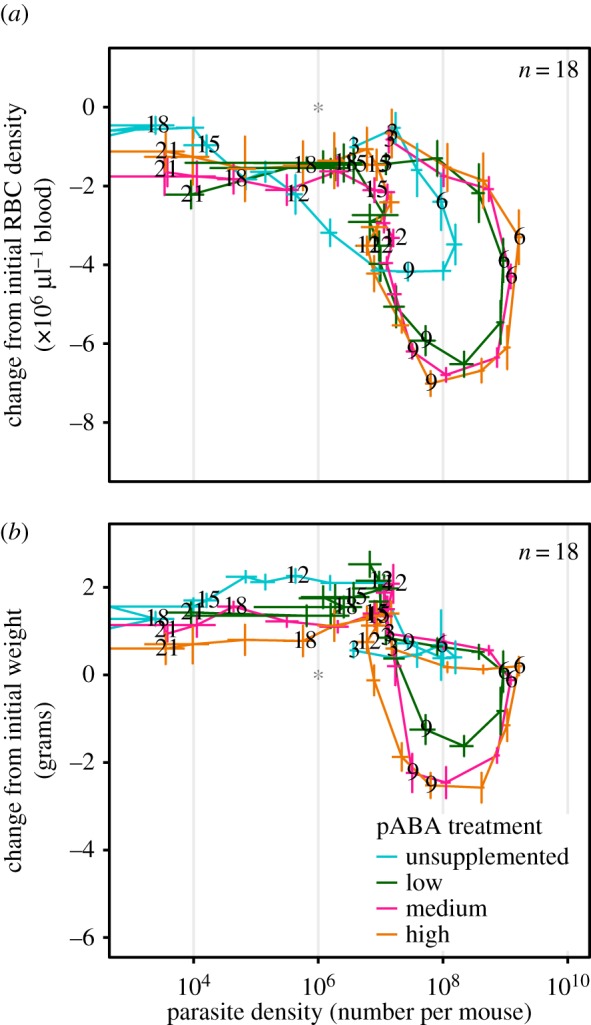
pABA supplementation alters the relationship between pathogen burden and disease in single infections. The relationship between parasite density and red blood cell (RBC) density (*a*) and parasite density and weight (*b*) through time in mice infected with AS_pyr_ only and given unsupplemented (blue) water or water supplemented with a low (green), medium (pink), or high (orange) concentration of pABA. Means and standard errors on each day, in each treatment, are connected by straight lines to form the trajectory. Numbers indicate the day of infection. Stars represent where in parasite-health space mice were on day 0.

The trajectory that mice took through ‘disease space’ [47,48] was affected by pABA (figure 4). Plotting time series data from experimental infections in health by microbe space helps to illuminate the (changing) relationship between pathology and pathogen burden and, in particular, the phase of the infection when the host is recovering [47]. *Plasmodium chabaudi* infected mice supplemented with the standard, high concentration of pABA, take a typical loop through pathogen-symptom ‘space’: mice initially remain healthy as parasite densities increase; as parasite densities reach their peak, mice begin to sicken; eventually, after parasite densities begin to fall, mice enter a recovery phase characterized by a reduction in both symptoms and parasite densities (figure 4, orange line; [48]). In mice infected with AS_pyr_ only pABA supplementation altered the relationship between parasite density and pathology: mice were more anaemic for a given parasite density during the first 10 days of infection and subsequently recovered to baseline red cell densities less rapidly than did unsupplemented mice (figure 4*a*). Mice supplemented with pABA recovered from infection differently from unsupplemented mice (figure 4*a*), recovering from anaemia before parasites numbered below 10 million. Similar patterns were seen for weight loss (figure 4*b*). In mixed infections, the impact of pABA treatment on the trajectories through disease space was much less pronounced (electronic supplementary material, figure S5*b*,*d*).

## 4. Discussion

Despite considerable interest in within-host interactions between parasite strains and their evolutionary consequences (e.g. [1,7,17,49]), and a slew of studies demonstrating that within-host competition occurs (e.g. [3–9]), the role of resources in mediating intraspecific competition has largely been the subject of speculation. Here, we experimentally demonstrate that pABA can mediate competition between strains of *P. chabaudi*, a biomedically relevant experimental system.

In single infections, pABA supplementation had a positive but saturating impact on the growth of AS_pyr_ consistent with its role as a limiting resource for AS_pyr_ [50] (figure 1; electronic supplementary material, figure S3). As malaria parasites use pABA for the production of pyrimidines for DNA synthesis [27], it seems probable that pABA supplementation promotes the growth of AS_pyr_ by increasing the numbers of offspring (merozoites) produced rather than reducing their susceptibility to immunity or the capacity to invade RBCs (in which parasites replicate). It is notable that, even in mixed infections, pABA did not alter the size of AJ infections (electronic supplementary material, figure S4). In several malaria parasite species, parasites resistant to pyrimethamine, like AS_pyr_, require more pABA for growth than drug susceptible parasites, like AJ, [30,32,51] possibly owing to the reduced capacity of pyrimethamine resistant parasites to acquire folate from molecules other than pABA [52]. Our data suggest that AS_pyr_ and AJ have similarly asymmetrical pABA requirements.

The asymmetrical resource requirements of AJ and AS_pyr_ could explain why, during the first 8 days of the infection, AS_pyr_ experienced competitive suppression more intensely in the high pABA treatment than in the other treatments (figure 2; electronic supplementary material, figure S3). This counterintuitive observation mirrors the finding in plant communities that the intensity of competition increases with environmental fertility and productivity [53,54]. The within-host environment of mice supplemented with low or medium concentrations of pABA may be one in which AJ and AS_pyr_ weakly interact: pABA concentrations are high enough that AJ's use of pABA does not strongly affect AS_pyr_, but AS_pyr_ remains primarily limited by pABA. In mice supplemented with a high concentration of pABA, however, AS_pyr_ is freed from pABA limitation and may begin to compete with AJ over access to other resources, such as RBCs, or via immune-mediated apparent competition, and so competition is more intense. It is possible that even in the unsupplemented treatment, pABA is not the substrate over which AJ and AS_pyr_ are *directly* competing—if, for example, pABA is not at a concentration limiting to AJ in unsupplemented mice. It has been suggested that there is a threshold population size below which parasites cannot escape the impact of immune killing [55]. The performance of AS_pyr_ in unsupplemented mice may be so poor that its population does not exceed this threshold, so that it suffers from intense immune-mediated apparent competition in unsupplemented mice. Of course, both immune-mediated competition and competition for pABA could be operating simultaneously.

Indeed, our data show that bottom-up and top-down forces can jointly regulate the population dynamics and competitive interactions of parasites and that the relative importance of these regulatory forces can change during an infection. The dynamics of *P. chabaudi* infections have been the focus of much theoretical attention (e.g. [20,55–62]). While it is generally accepted that the immune response is responsible for the post-peak control of malaria infections [20,55,57,63–65], there has been intense debate about the forces that regulate the dynamics of parasite populations prior to their clearance. Both RBC availability and the immune response have been invoked to explain the growth rate and peak density of infections [20,55,57,59]; similarly, differences in the way that strains interact with the immune response or RBCs have been proposed to explain the dynamics of interstrain competition [20,59,61]. The hypothesis that RBCs mediate interstrain competition has gone untested while the role of immune-mediated apparent competition has received mixed empirical support [22,23]. Our data represent, to our knowledge, the first experimental demonstration that resource availability determines the growth rate, size and timing of peak density of malaria infections and the intensity of interstrain competition between parasite strains. These data suggest that resources may play a vital role in determining the dynamics of malaria infections prior to the onset of adaptive immunity, which is responsible for the clearance of infection. Rarely have models that focus on a single dimension of the niche been sufficient to explain either the dynamics or diversity of non-parasitic populations; given the complexity of the within-host environment it would be surprising if they sufficed to explain all infection dynamics**.** Our work attests to the use of broadening our conception of the parasite niche beyond the two axes of target cells and immunity.

That the relationship between parasite burden and disease changes with pABA supplementation (figure 4), suggests that resource supply may not only act in concert with immunity or other resources to govern infection dynamics, but may also change the nature or role of these other regulatory factors. Supplementation of pABA causes an increase in the severity of disease associated with a given number of parasites in the first 10 days of infection (tolerance [66]), a qualitative shift in the process of recovery from infection (figure 4) and a hump in the infection dynamics in the post-peak period (figure 1), during which the dynamics of infection are thought to be governed by the immune system [20,55,57,63–65]. It is unlikely that pABA has a direct impact on the host's response to infection, since mice do not require it [67] and removing pABA from the host's diet has a similar impact on parasite growth in intact mice as in immune-deficient mice [33]. Instead, by altering parasite traits (i.e. growth rate) to which the host responds, pABA supplementation could cause a qualitative change in the host immune or erythropoietic response. pABA supplementation may indirectly cause a switch to a more immunopathologic response. This hypothesis would account for the increase in virulence observed with pABA supplementation (figure 3), since the loss of both weight and RBCs in malaria infection is caused by the activity of the immunopathologic, TH1 arm of the immune system [65,68]. The possibility that the availability of a parasite nutrient alters host tolerance warrants further investigation. Indeed, experimental manipulations of pABA could be used to learn about the host-parasite interaction, in addition to parasite-parasite interactions.

It is tempting to ask whether pABA's availability might be manipulated for the promotion of patient health. The original observations that pABA limited the growth of malaria parasites led to speculation that putting malaria-infected patients on dairy-based diets, which contain very low concentrations of pABA, could be used to limit their parasitaemia [29,69]. Our data indicates that the success of such manipulations would depend on infection composition and/or the dependence of individual strains upon pABA. Dietary manipulation of pABA may also be used to promote patient health indirectly, by improving and preserving the efficacy of antifolate drug treatment. *In vitro*, the efficacy of sulfadoxine/pyrimethamine (S/P) drug treatment is inversely related to the concentration of pABA in the medium, because pABA competes with sulfadoxine (a pABA analogue) for its dihydropteroate synthase binding site [70–72]. Kicska *et al*. [33] proposed that diets low in pABA be administered to patients receiving S/P treatment, to boost S/P's efficacy. With S/P being the only drug approved for prophylactic treatment of pregnant women, and resistance to it prevalent, such an intervention could be of value.

Manipulations of pABA availability could also play a role in slowing the evolution of drug resistance, since competitive interactions are at the heart of the process of drug resistance evolution [15,16] and pABA mediates these competitive interactions. Susceptible pathogens competitively suppress resistant pathogens, as was observed here; drug treatment removes these competitors, allowing resistant parasites to flourish [15]. Reducing the availability of pABA in the host environment could be used to intensify the competitive suppression of drug resistant parasites in the period before susceptible parasites have been cleared by the drug, reducing the probability that resistant parasites will survive to emerge once released from competition. It is intriguing to think that we may have altered competitive interactions between malaria parasites for years, unwittingly, via our administration of sulfadoxine treatment, which blocks parasites' access to pABA. Resource depletion may be a relatively ‘evolution-proof’ strategy, as compared to giving a drug that blocks access to a resource, since parasites would not be able to employ common resistance mechanisms such as efflux pumps or target site mutations to resist it. The extent to which pABA manipulation can be used to manage resistance will depend on the extent to which malaria parasites in the field are limited by pABA, a question open for further investigation.

## Supplementary Material

Figure S1: Dynamics of single infections in individual mice

## Supplementary Material

Figure S2: Dynamics of mixed infections in individual mice

## Supplementary Material

Figure S3: The impact of pABA treatment on infection size in single and mixed infections

## Supplementary Material

Figure S4: In mixed infections, pABA treatment has a minimal impact on the growth of AJ

## Supplementary Material

Figure S5: The impact of pABA supplementation on the relationship between pathogen burden & disease in single and mixed infections

## Data accessibility

The dataset supporting this article is available from the Dryad Digital Repository: http://dx.doi.org/10.5061/dryad.f502p [73].
