# Supplementary figures and images for: A nutrient mediates intraspecific competition between rodent malaria parasites in vivo
Source: Proc Biol Sci. 2017 Jul 26;284(1859):20171067. doi: 10.1098/rspb.2017.1067 (PMC5543226; doi:10.1098/rspb.2017.1067)

# Single Infections

Density of  $AS_{pyr}$  (Number per Mouse)

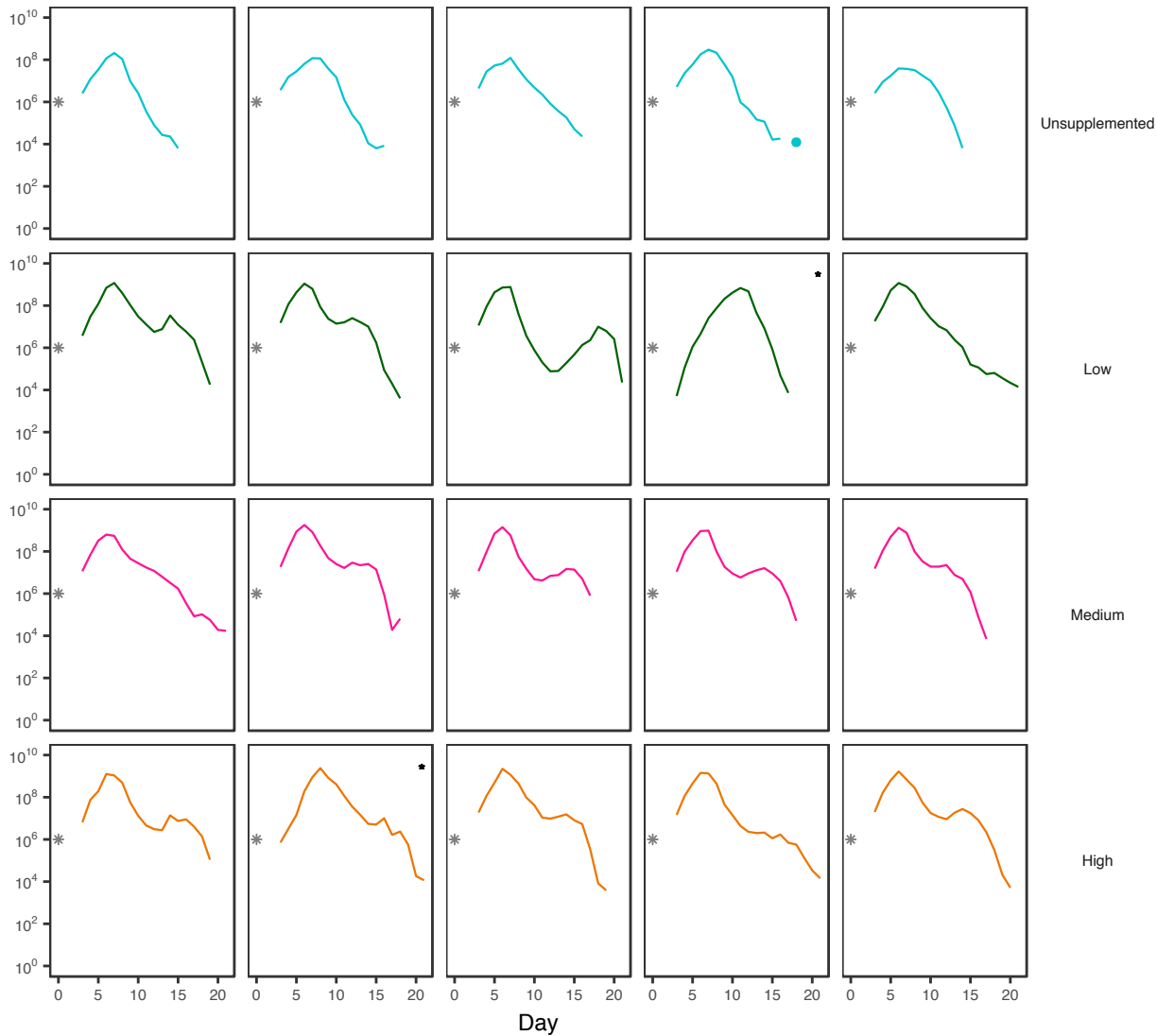

Supplement: Figure S1: Dynamics of single infections in individual mice [file rspb20171067supp1.pdf]

# Mixed Infections

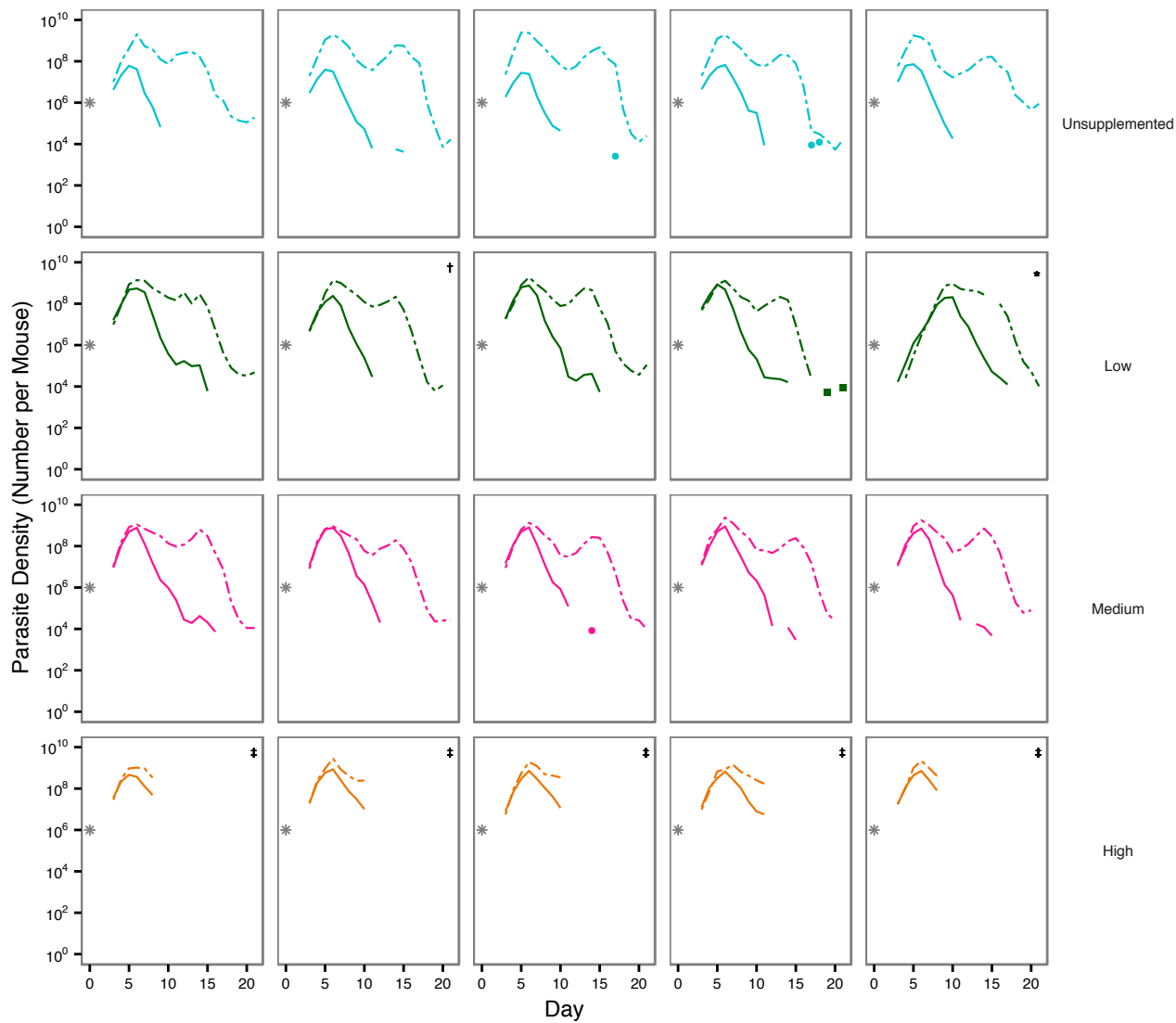

Supplement: Figure S2: Dynamics of mixed infections in individual mice [file rspb20171067supp2.pdf]

Total Density of AS<sub>pyr</sub> (Number per Mouse)

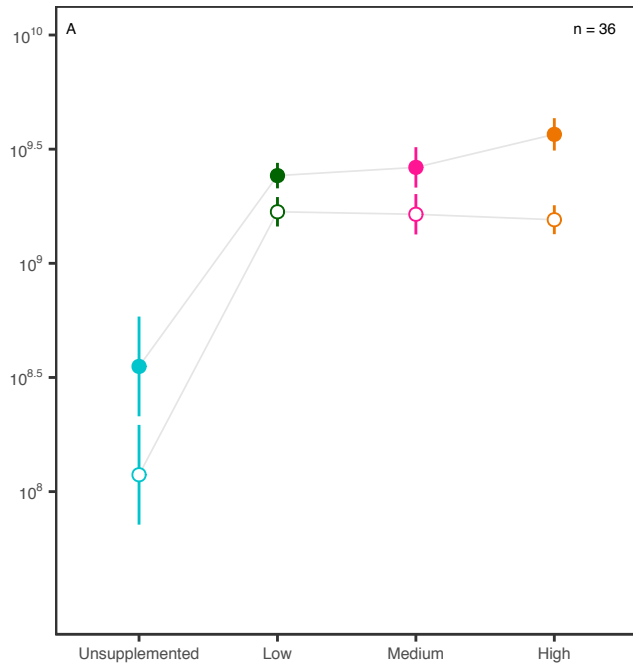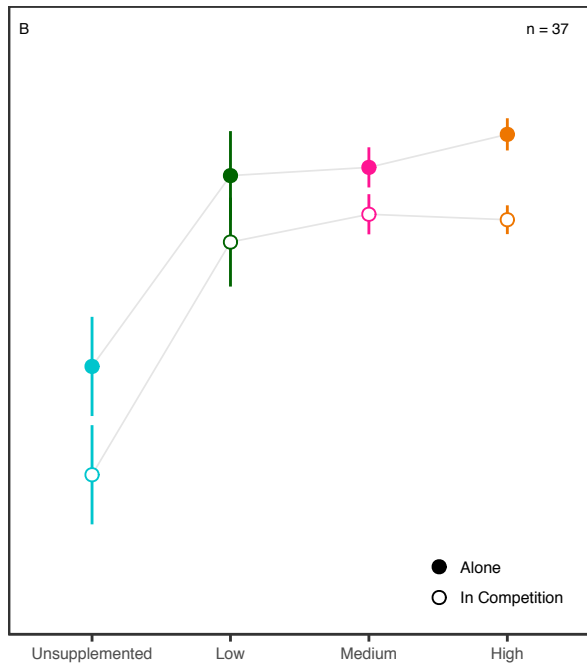

pABA Treatment

Supplement: Figure S3: The impact of pABA treatment on infection size in single and mixed infections [file rspb20171067supp3.pdf]

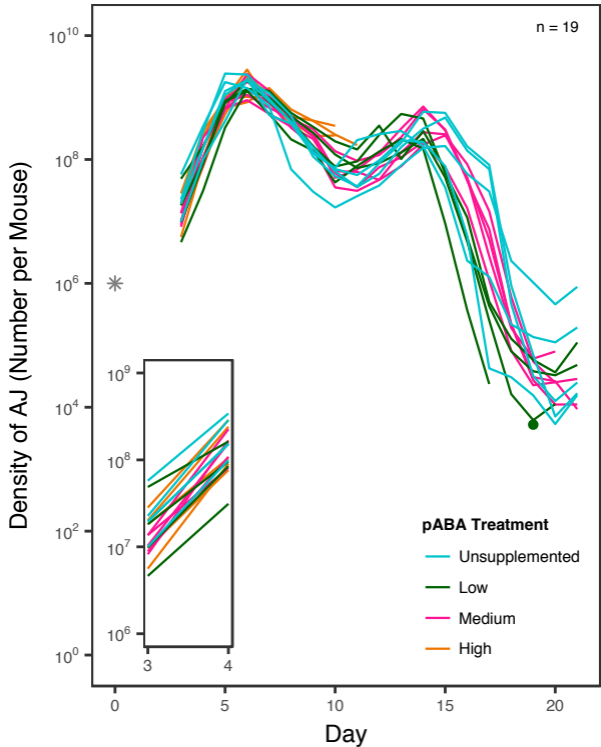

Supplement: Figure S4: In mixed infections, pABA treatment has a minimal impact on the growth of AJ [file rspb20171067supp4.pdf]
