# Supplementary material for: A nutrient mediates intraspecific competition between rodent malaria parasites in vivo
Source: Proc Biol Sci. 2017 Jul 26;284(1859):20171067. doi: 10.1098/rspb.2017.1067 (PMC5543226; doi:10.1098/rspb.2017.1067)
Supplement: Figure S5: The impact of pABA supplementation on the relationship between pathogen burden & disease in single and mixed infections [file rspb20171067supp5.pdf]

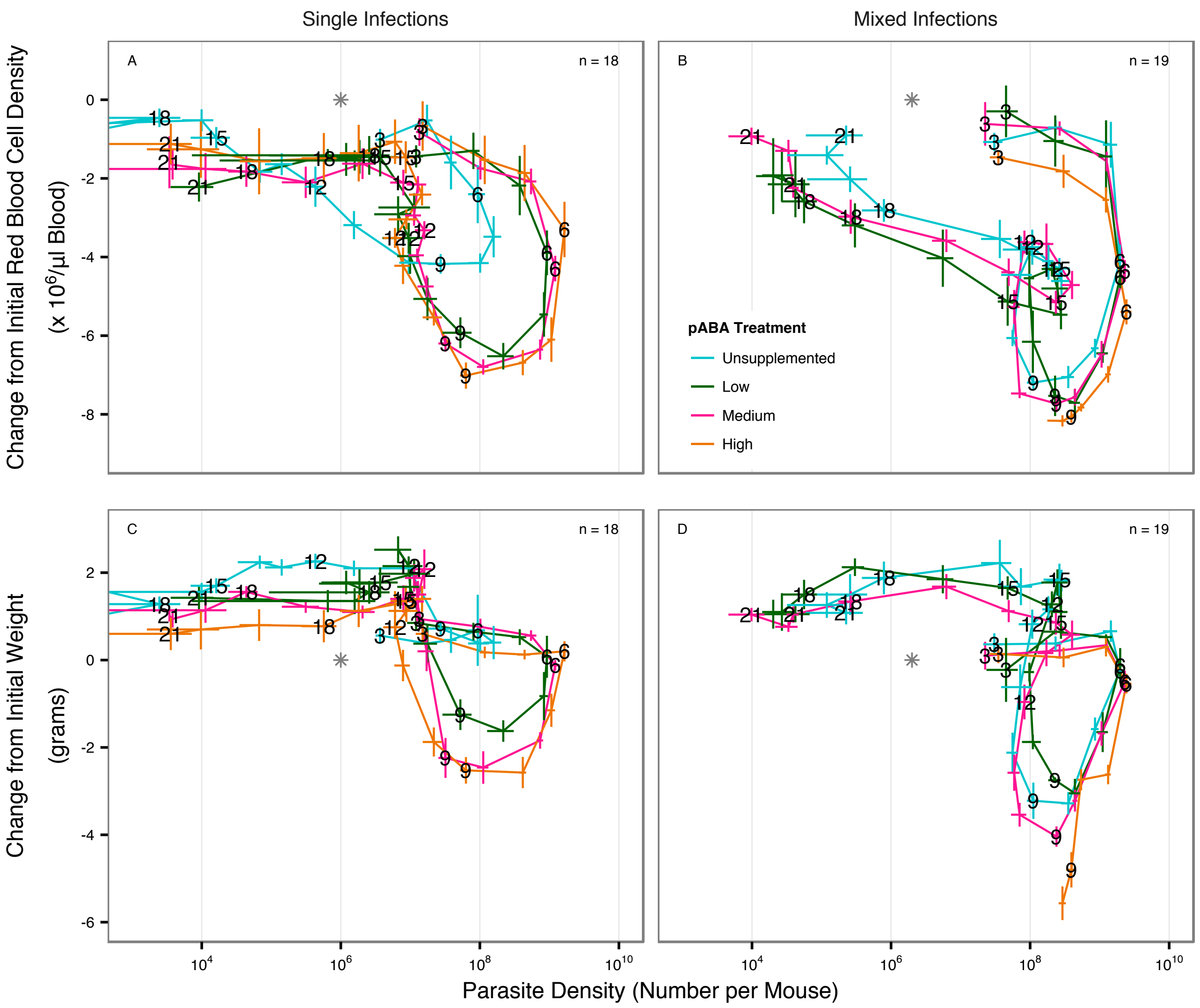

Single Infections
Mixed Infections
Change from Initial Red Blood Cell Density
(x 10^6/µl Blood)
Change from Initial Weight
(grams)
A
n = 18
B
n = 19
C
n = 18
D
n = 19
pABA Treatment
Unsupplemented
Low
Medium
High
Parasite Density (Number per Mouse)
